# Corrigendum to “SANDI: A compartment-based model for non-invasive apparent soma and neurite imaging by diffusion MRI” [Neuroimage 215 (2020), 116835]

**DOI:** 10.1016/j.neuroimage.2020.117612

**Published:** 2021-02-01

**Authors:** Marco Palombo, Andrada Ianus, Michele Guerreri, Daniel Nunes, Daniel C. Alexander, Noam Shemesh, Hui Zhang

**Affiliations:** a Centre for Medical Image Computing and Dept of Computer Science, University College London, London, UK; b Champalimaud Research, Champalimaud Centre for the Unknown, Lisbon, Portugal

The authors regret that due to an error in the code used to process the data, the estimated parametric maps reported in Figure 9 are incorrect. The corrected Figure 9 is included here.

**Figure 9:**
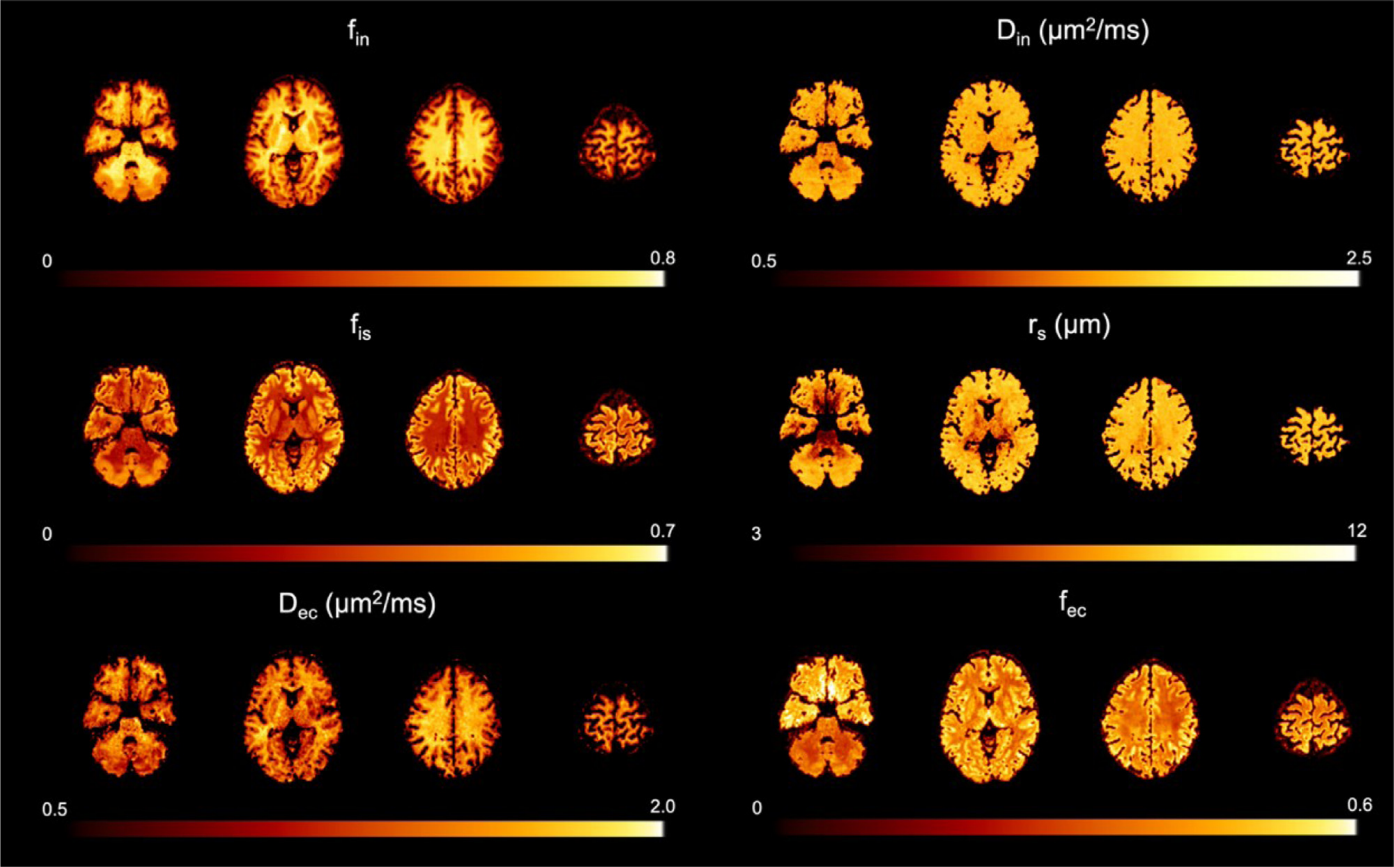
An example of the parametric maps of the proposed compartment model for brain microstructure, obtained by fitting Eq. (10) to the normalised direction-averaged DW-MRI data from a representative subject.

The primary differences to the original Figure 9 are in the estimated parametric maps D_in_, D_ec_ and f_ec_. However, the observed contrast in f_in_, f_is_ and r_s_ is minimally affected and remains compatible with the histological counterparts shown in Figure 10-12, hence the main conclusions still hold.

Furthermore, it has been brought to the authors’ attention that our statement about the adequacy of the MGH Adult Diffusion Dataset is incorrect. The statement drew inference from the results of the ablation study shown in Figure 8, which considers *only* the simulated intra-cellular signal (i.e. we assume f_ec_ = 0; thus, f_ec_ and D_ec_ are not estimated, leaving only three free parameters). The in vivo human data have additional signal contributions from the extra-cellular space (f_ec_ > 0 so f_ec_ and D_ec_ become relevant and the number of free parameters increases to five), which our simulations do not reflect. Thus, indeed, the conclusions may not extend to the in vivo human data and we would like to correct the following statement:

• The original paragraph on page 10 (second column, third paragraph) reads: ‘*The ablation study is reported in Fig. 8, showing that a minimum of five b values (or b shells), with two of them higher than 3,000 ​s/mm*^*2*^*(or equivalently 3 ​ms/μm*^*2*^*) are required to produce parameter estimates of reasonable accuracy and precision. These results suggest that the in vivo human dataset used in this work (MGH Adult Diffusion Dataset) is adequate (third column in Fig. 8), but less and/or lower b values would be insufficient, which would lead to MSE values 2 to 30 times larger (first and second column in Fig. 7).*’
• The corrected paragraph should read: ‘*The ablation study is reported in Fig. 8, showing that having four or more nonzero b values (or b shells), with two of them higher than 3,000 ​s/mm^2^ (or equivalently 3 ​ms/μm^2^), produces parameter estimates of reasonable accuracy and precision; but having less than two b values (or b shells) higher than 3,000 ​s/mm^2^ (or equivalently 3 ​ms/μm^2^) may be insufficient and can lead to MSE values 2 to 30 times larger (first and second column in Fig. 8).*’

This highlights further that the results in Figure 9 come ostensibly from fitting five free parameters to a data set with only four independent data points (four nonzero b-shells). Despite the apparent underdetermination, the fitting procedure still provides robust estimates of four parameters (D_ec_, f_ec_, f_in_, and r_s_) because the data has little or no sensitivity to D_in_ and the random-forest regressor implicitly fixes it to a value close to the average of the settings in the training data. An additional sensitivity analysis reported here in Figure X1 confirms this and shows that in fact adding more independent data points with intermediate b-values has little effect on the estimated parameters. This observation prompts an additional correction to the original manuscript:

• The original paragraph on page 17 (first column, third paragraph, last sentence) reads: *’The ablation study demonstrates that the MGH Adult Diffusion Dataset used in this work is an example of a minimal dataset that meets these requirements: short enough diffusion time, high enough b values, six b-shells to estimate five model parameters.’*
• The corrected paragraph should read: ‘*The ablation study demonstrates that a minimal dataset should meet these requirements: short enough diffusion time, high enough b values. Of course the dataset must also have at least as many nonzero b-values as free parameters: three or more nonzero b-shells to estimate the three model parameters (D_in_, f_in_, and r_s_) when f_ec_ = 0 as in the ablation study; five or more for real data where f_ec_ is typically nonzero, although fewer b-shells can still provide viable estimates if for example, as in the human data in* Figure 9*, the fitting procedure implicitly fixes D_in_ to reasonable values.*’

**Figure X1:**
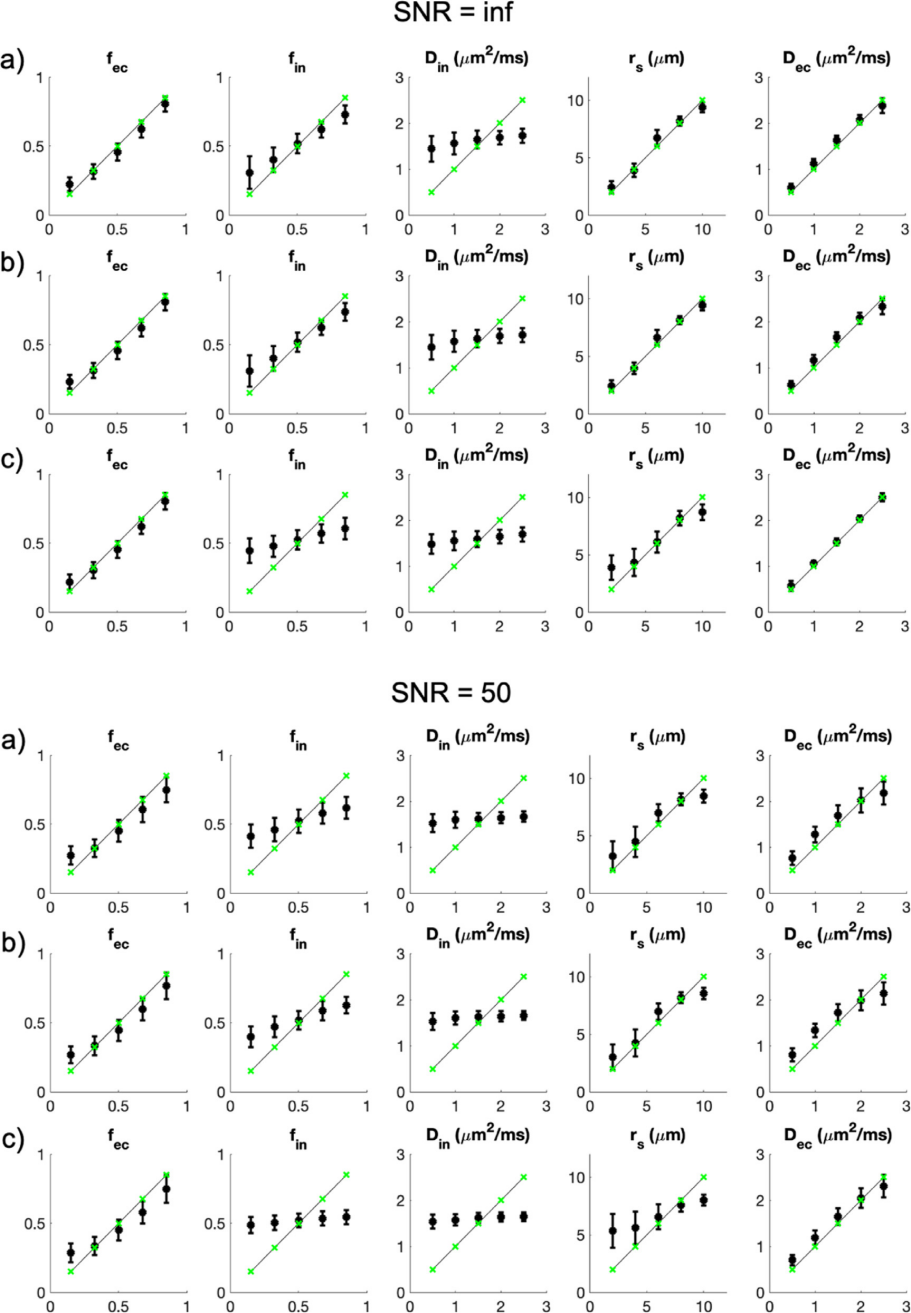
Sensitivity analysis showing the estimated (y-axis) versus ground truth (x-axis) values of the five SANDI model parameters. We added Rician noise corresponding to SNR = inf and 50 to signals simulated using equation (10) and 3125 combinations of the model parameters obtained from five values linearly spaced in the intervals: f_ec_=[0.15,0.85]; f_in_=[0.15,0.85]; D_in_=[0.5,2.5] µm^2^/ms; r_s_=[2,10] µm; D_ec_=[0.5,2.5] µm^2^/ms. We report results from three protocols: a) the original MGH Adult Diffusion Dataset: Δ/δ=22/13 ms, b=[0,1,3,5,10] ms/µm^2^; b) a richer protocol: Δ/δ=22/13 ms, b=[0,1,2,3,4,5,6,7,8,9,10] ms/µm^2^; c) a protocol achievable on clinical scanners: Δ/δ=22/13 ms, b=[0,0.5,0.75,1.2,1.5,2,2.5] ms/µm^2^. Black datapoints are the median values of the estimated parameters for the different combinations; error bars are their quartile deviations. Green data points are the ground truth values and the solid lines are the identity lines.

We emphasise that the main conclusions of the original manuscript are not affected by these issues, since they are drawn from the results of the detailed numerical simulations and the rich mouse dataset; the human data are included simply as a preliminary proof-of-concept for future work.

The authors would like to apologise for any inconvenience caused.

