# Supplementary material for: SANDI: A compartment-based model for non-invasive apparent soma and neurite imaging by diffusion MRI
Source: Neuroimage. 2020 Jul 15;215:116835. doi: 10.1016/j.neuroimage.2020.116835 (PMC8543044; doi:10.1016/j.neuroimage.2020.116835)
Supplement: Multimedia component 1 [file mmc1.docx]

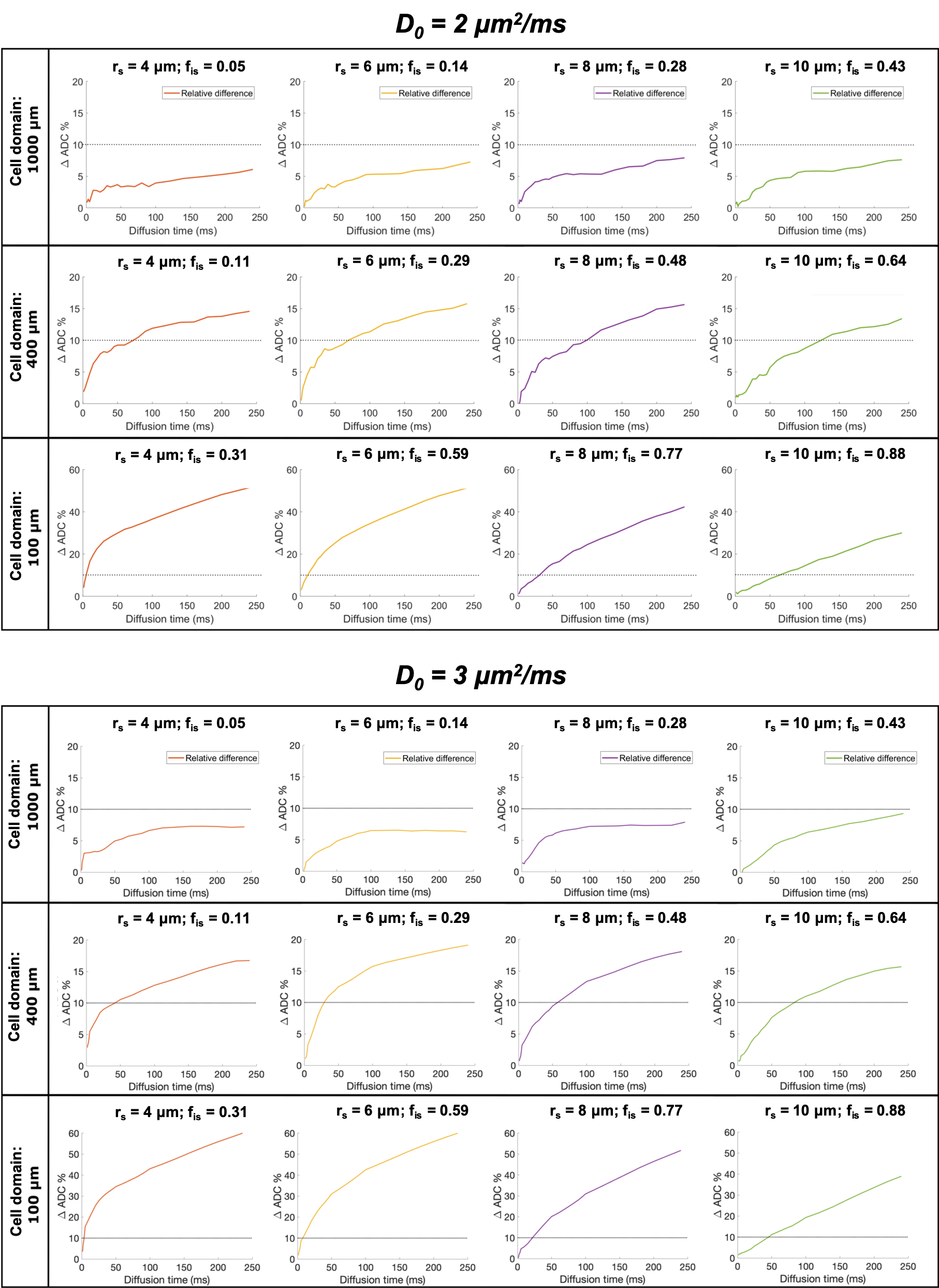


**Figure S1**. Regime of validity of the compartment model for different bulk diffusivities. Relative percentage difference between the ADC in the exchange and non-exchange cases with bulk diffusivity D_0_ = 2 μm^2^/ms (like in **Figure 3**) and D_0_ = 3 μm^2^/ms. The dashed lines show the 10% threshold used to define the diffusion time regime where the compartment model (10) is a reasonable approximation of cellular structures.


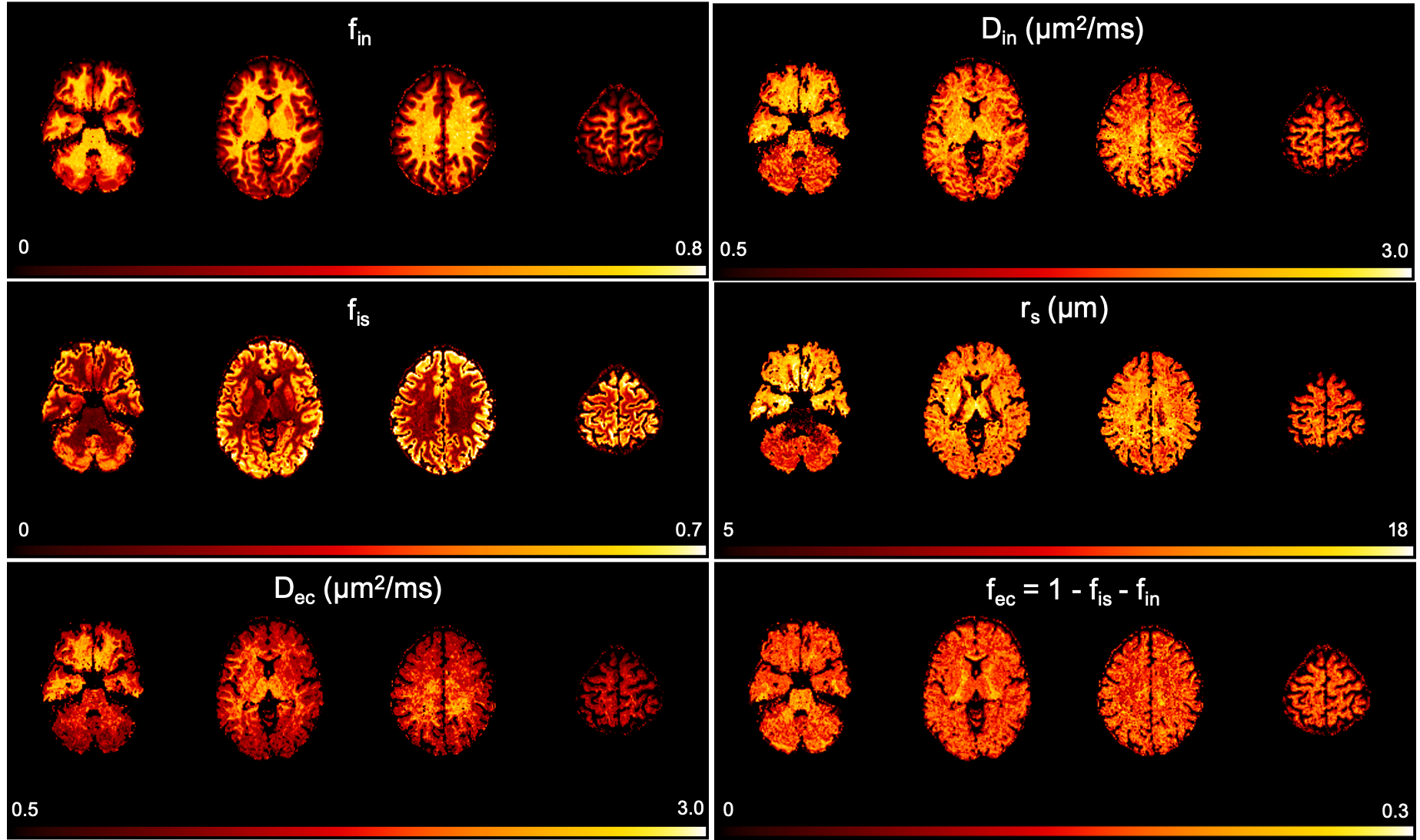


**Figure S2**. Example of SANDI parametric maps obtained by using a different training set from the one chosen in the Methods, section 3.3. In this case, the random forest regressor was trained using the following intervals for the model parameters: $f_{in}$=[0.01, 0.99]; $f_{ec}$=[0.01, 0.99]; $D_{in}$=[0.1, 3] μm^2^/ms; $D_{ec}$=[0.1, 3] μm^2^/ms; $r_{s}$=[1, 20] μm. Given the specific experimental protocol used in this study (t_d_~20 ms), the measured DW-MRI signal is sensitive to soma size $r_{s}$ ≤ 12 μm. Therefore, training with $r_{s}$=[1, 20] μm is incorrect and leads to lower accuracy and precision in the model parameters prediction, especially for $r_{s}$, $D_{in}$ and $D_{ec}$, as evident from the comparison with **Figure 9**. In fact, we would expect lower $r_{s}$ in WM than in GM regions, and more uniform $D_{in}$ and $D_{ec}$ within WM and GM regions. While this is the case in **Figure 9**, it is clearly not the case here.
